# Supplementary material for: Increases in Arctic Extreme Climatic Events Are Linked to Negative Fitness Effects on the Local Biota
Source: Glob Chang Biol. 2025 Apr 1;31(4):e70157. doi: 10.1111/gcb.70157 (PMC11960799; doi:10.1111/gcb.70157)
Supplement: Supplementary file 1 — Data S1. [file GCB-31-e70157-s001.pdf]

Appendices

All R scripts and data files are available online at: https://doi.org/10.5281/zenodo.15011515

Appendix 1 – The 'Arctic Region'

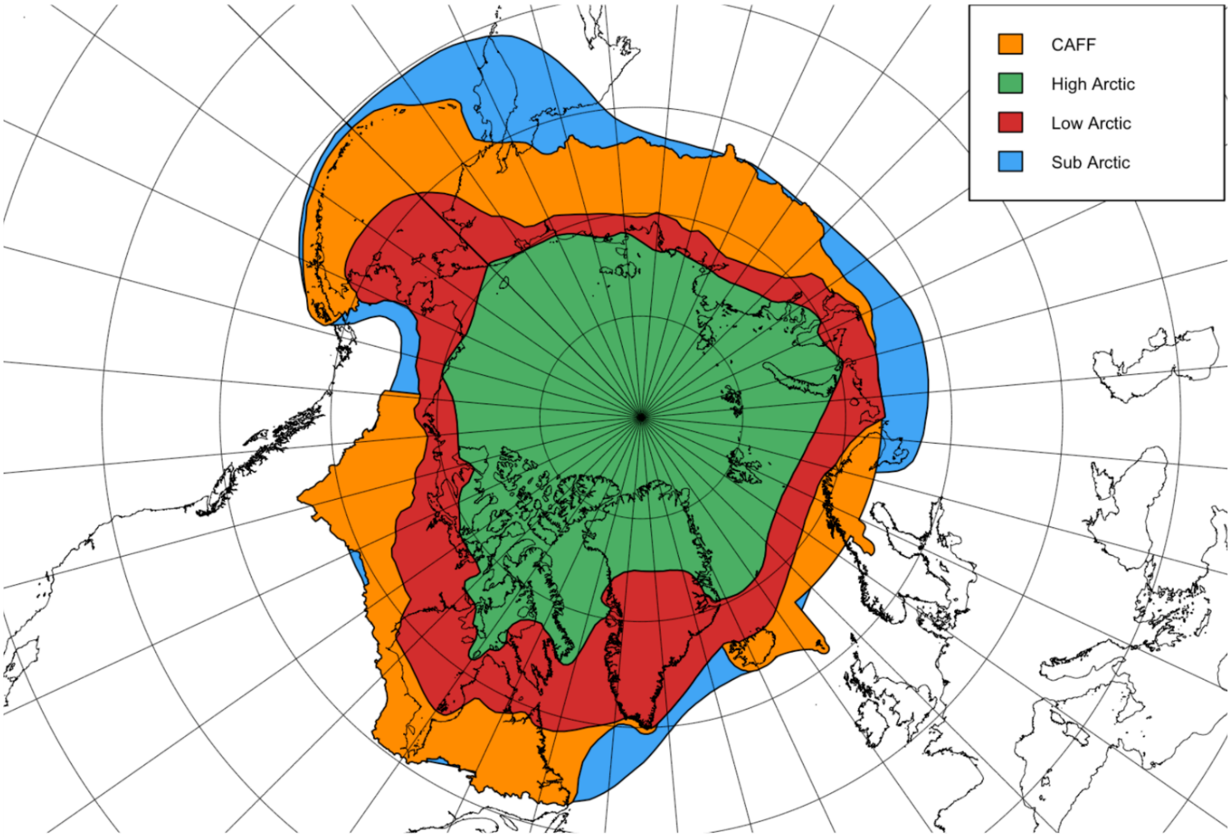

The Arctic region adapted from Martin *et al.*, (2022), the maximum extent of which has been used to define the Arctic in this study (i.e., the outer contour of all coloured regions). Orange = Conservation of Arctic Flora and Fauna (CAFF) boundary, Green = Arctic Biodiversity Assessment (ABA): High Arctic, Red = ABA: Low Arctic, Blue = ABA: Sub Arctic.

Appendix 2 – Table of most frequently used words

Word	Frequency
**arctic**	1628
**chang**	1542
**event**	1268
**climat**	1249
**temperatur**	1249
**winter**	1175
**speci**	1120
**warm**	1107
year	1094
use	990
plant	973
effect	963
soil	941
increas	883

Word	Frequency
studi	876
plot	869
snow	812
**popul**	766
**extrem**	758
veget	748
high	719
**tundra**	713
site	696
may	695
data	694
ice	689
area	677
fig	671

Word	Frequency
condit	665
model	633
differ	624
growth	619
**ecolog**	609
season	608
respons	599
summer	591
time	573
**ecosystem**	559
caribou	552
observ	527
**weather**	525
cover	522

The 42 most frequent words used in the 10 relevant papers identified in the scoping search to identify words for inclusion in the search string of our meta-analysis. Pertinent words were used to produce a comprehensive search string with words in bold being selected for inclusion in the finalised search string.

Appendix 3 – Fine-tuning and final search strings

Fine-tuning of the search string led to the addition of “NOT (human OR energy OR infrastructure)” to each search string due to the high number of human health, infrastructure, and energy-related papers not relevant to our research questions.

The final search string for Scopus was:

TITLE-ABS-KEY (("extreme event" OR "extreme climat* event" OR "extreme weather" OR "extreme heat" OR "extreme temperature" OR "extreme precipitation" OR "rain on snow" OR "extreme winter warming") AND (tundra OR arctic) AND (ecosystem OR ecology OR species OR populations) AND NOT (human OR energy OR infrastructure)).

The final search string for Web of Science was:

TI = (("extreme event" OR "extreme climat* event" OR "extreme weather" OR "extreme heat" OR "extreme temperature" OR "extreme precipitation" OR "rain on snow" OR "extreme winter warming") AND (tundra OR arctic)) OR AB = (("extreme event" OR "extreme climat* event" OR "extreme weather" OR "extreme heat" OR "extreme temperature" OR "extreme precipitation" OR "rain on snow" OR "extreme winter warming") AND (tundra OR arctic) AND (ecosystem OR ecology OR species OR populations) NOT (human OR energy OR infrastructure)).

Appendix 4 - List of 17 papers included in meta-analysis (data sources)

1. Bjerke, J. W. *et al.* Persistent Reduction of Segment Growth and Photosynthesis in a Widespread and Important Sub-Arctic Moss Species After Cessation of Three Years of Experimental Winter Warming. *Functional Ecology* **31**, 127–134 (2017). https://doi.org/10.1111/1365-2435.12703

2. Bokhorst, S. *et al.* Impacts of Extreme Winter Warming in the Sub-Arctic: Growing Season Responses of Dwarf Shrub Heathland. *Global Change Biology* **14**, 2603–2612 (2008). https://doi.org/10.1111/j.1365-2486.2008.01689.x

3. Bokhorst, S. *et al.* Warming Events Damage Sub-Arctic Vegetation: Consistent Evidence From an Experimental Manipulation and a Natural Event. *Journal of Ecology* **97**, 1408–1415 (2009). https://doi.org/10.1111/j.1365-2745.2009.01554.x

4. Bokhorst, S. *et al.* Impacts of Extreme Winter Warming Events on Litter Decomposition in a Sub-Arctic Heathland. *Soil Biology and Biochemistry* **42**, 611–617 (2010a). https://doi.org/10.1016/j.soilbio.2009.12.011

5. Bokhorst, S. *et al.* Impacts of Multiple Extreme Winter Warming Events on Sub-Arctic Heathland: Phenology, Reproduction, Growth, and CO2 Flux Responses. *Global Change Biology* **17**, 2817–2830 (2011). https://doi.org/10.1111/j.1365-2486.2011.02424.x

6. Bokhorst, S. *et al.* Extreme Winter Warming Events More Negatively Impact Small Rather Than Large Soil Fauna: Shift in Community Composition Explained by Traits Not Taxa. *Global Change Biology* **18**, 1152–1162 (2012a). https://doi.org/10.1111/j.1365-2486.2011.02565.x

7. Bokhorst, S. *et al.* Vegetation Recovery Following Extreme Winter Warming Events in the Sub-Arctic Estimated Using NDVI From Remote Sensing and Handheld Passive Proximal Sensors. *Environmental and Experimental Botany* **81**, 18–25 (2012b). https://doi.org/10.1016/j.envexpbot.2012.02.011

8. Bokhorst, S. *et al.* Climatic and Biotic Extreme Events Moderate Long-Term Responses of Above- and Belowground Sub-Arctic Heathland Communities to Climate Change. *Global Change Biology* **21**, 4063–4075 (2015). https://doi.org/10.1111/gcb.13007

9. Bokhorst, S. *et al.* Contrasting Survival and Physiological Responses of Sub-Arctic Plant Types to Extreme Winter Warming and Nitrogen. *Planta* **247**, 635–648 (2018). https://doi.org/10.1007/s00425-017-2813-6

410. Bokhorst, S. *et al.* Sub-Arctic Mosses and Lichens Show Idiosyncratic Responses to Combinations of Winter Heatwaves, Freezing and Nitrogen Deposition. *Physiologia Plantarum* **175**, (2023). https://doi.org/10.1111/ppl.13882
11. Hansen, B. B. *et al.* Climate Events Synchronize the Dynamics of a Resident Vertebrate Community in the High Arctic. *Science* **339**, 313–315 (2013). https://doi.org/10.1126/science.1226766
12. Loe, L. E. *et al.* Behavioral Buffering of Extreme Weather Events in a High-Arctic Herbivore. *Ecosphere* **7**, (2016). https://doi.org/10.1002/ecs2.1374
13. Milner, J. M. *et al.* Experimental Icing Affects Growth, Mortality, and Flowering in a High Arctic Dwarf Shrub. *Ecology and Evolution* **6**, 2139–2148 (2016). https://doi.org/10.1002/ece3.2023
14. Poirier, M. *et al.* Snow Hardness Impacts Intranivean Locomotion of Arctic Small Mammals. *Ecosphere* **12**, (2021). https://doi.org/10.1002/ecs2.3835
15. Semenchuk, P. R. *et al.* Snow Cover and Extreme Winter Warming Events Control Flower Abundance of Some, but Not All Species in High Arctic Svalbard. *Ecology and Evolution* **3**, 2586–2599 (2013). https://doi.org/10.1002/ece3.648
16. Stien, A. *et al.* Congruent Responses to Weather Variability in High Arctic Herbivores. *Biology Letters* **8**, 1002–1005 (2012). https://doi.org/10.1098/rsbl.2012.0764
17. Treharne, R. *et al.* Arctic Browning: Impacts of Extreme Climatic Events on Heathland Ecosystem CO2 Fluxes. *Global Change Biology* **25**, 489–503 (2019). https://doi.org/10.1111/gcb.14500

Appendix 5 - Categorisation of response variables into two broader categories

Quantity	Energetic requirements
Abundance	Tunnel length (burrowing depth)
Peak flower abundance	Time to layer B (burrowing depth)
Alive:dead shoots ratio	Time to layer C (burrowing depth)
Biomass (above ground density)	Displacement (migration)
Shoot growth	PSII activity
Calf at heel (proportion of females with calf)	Photosynthetic rate
Winter mortality (standardised)	
April body mass (biomass)	
Berry production	
Survival	
Shoot alive	
Greenness	
Segment length	
Segment width	
Length:width ratio	

Response variables used in our meta-analysis to determine whether rain-on-snow and extreme winter warming events in the Arctic have a significant negative effect on the fitness of native biota, categorised into two broader categories to allow for comparisons across the 17 studies examined. Quantity-related variables assess changes in population and individual traits, while energetic requirements examine physiological and metabolic adjustments resulting from the consequences of extreme winter events.

Appendix 6 – Arctic regions have experienced a significant increase in average maximum daily winter temperature

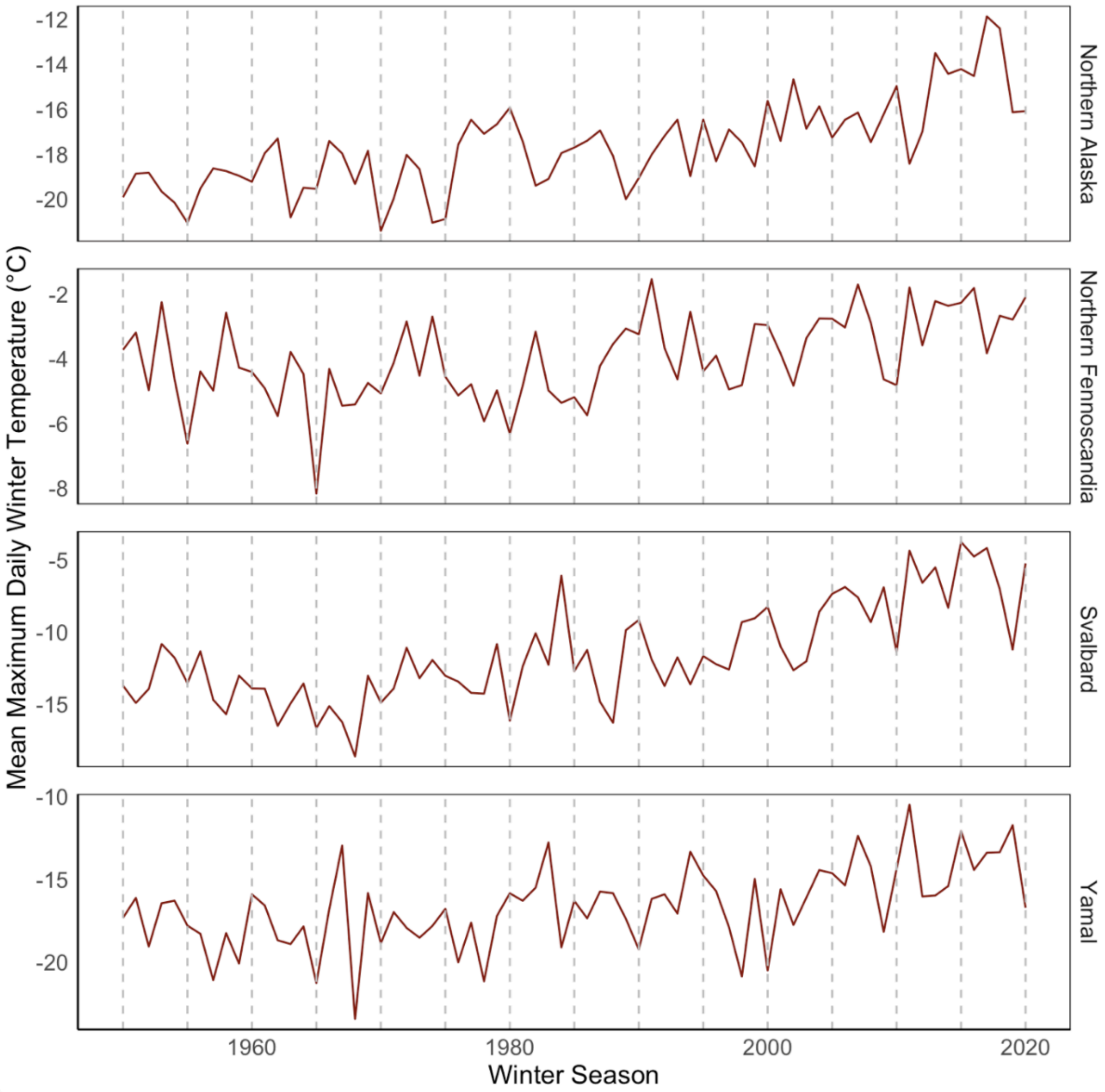

Mean maximum daily winter temperatures (°C) averaged across different Arctic areas of special interest (see Methods) across winter seasons (October to March). Grey dotted lines represent 5-year intervals. Areas of special interest are ordered alphabetically from top to bottom: Northern Alaska, Northern Fennoscandia, Svalbard, and Yamal (North-western Siberia).

Appendix 7 – Most Arctic regions have experienced an increase in annual precipitation since 1940

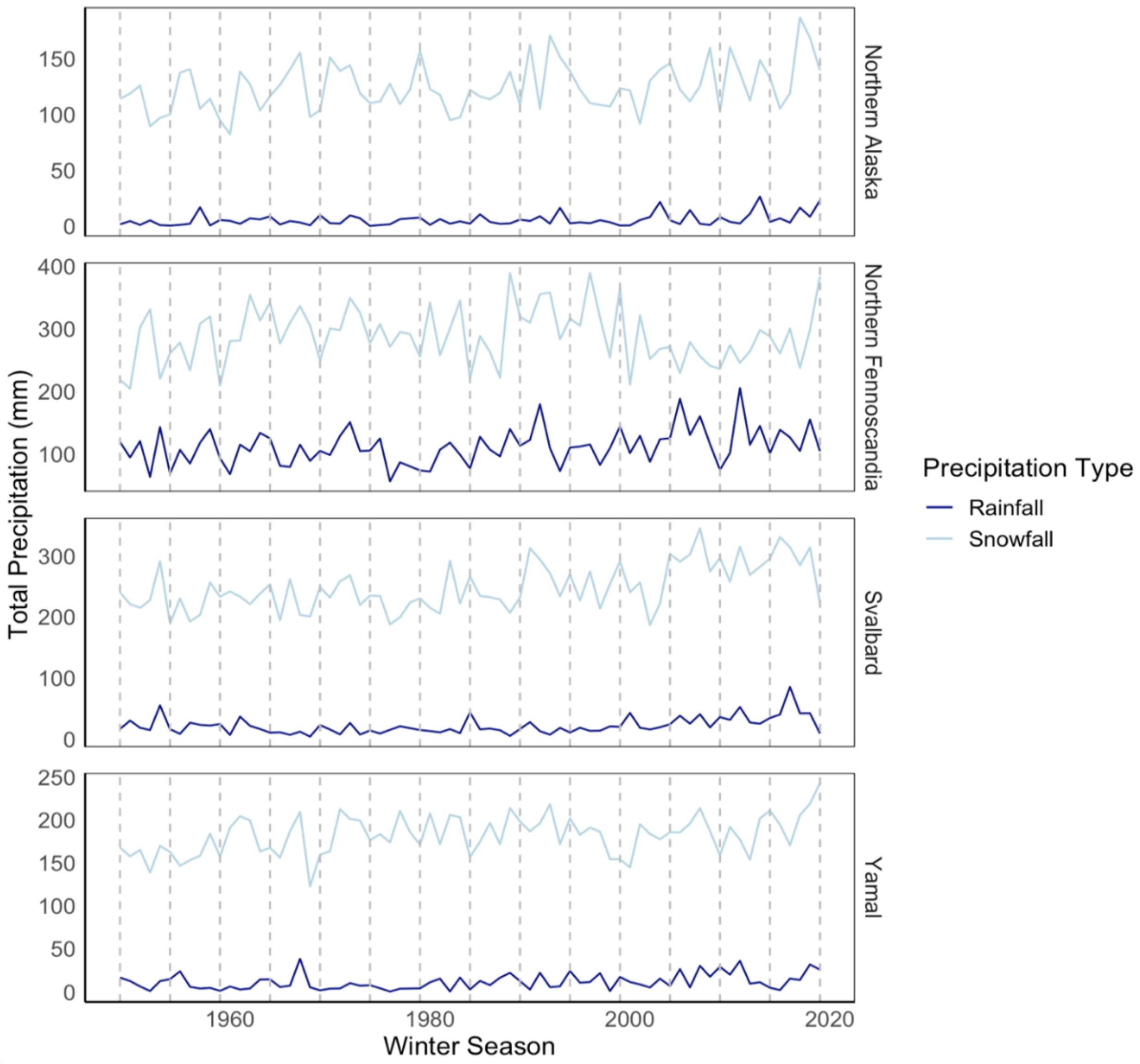

Total snowfall in millimetres water equivalent (light blue) and rainfall in millimetres (dark blue)in each winter season (October to March) for the Arctic areas of special interest (see Methods) from 1950-2020. Grey dotted lines represent 5-year intervals. Regions are ordered alphabetically from top to bottom: Northern Alaska, Northern Fennoscandia, Svalbard, and Yamal (North-western Siberia).

Appendix 8 – Heterogeneity explained in the models with different moderators

**Model**	$R^2 (\%)$
Full	6.66
Experimental Y/N	1.27
Event Type	3.41
Kingdom	1.87
Phylum	7.23
Taxonomic class	6.76
Broad Category	0.02
